# Contribution of Osteoblast and Osteoclast Supernatants to Bone Formation: Determination Using a Novel Microfluidic Chip

**DOI:** 10.3390/ijms25126605

**Published:** 2024-06-15

**Authors:** Sin Hyung Park, Hyun-Ju An, Haeri Kim, Insun Song, Soonchul Lee

**Affiliations:** 1 Department of Orthopaedic Surgery, Bucheon Hospital, Soonchunhyang University School of Medicine, 170 Jomaru-ro, Bucheon-si 14584, Gyeonggi-do, Republic of Korea; greatpsh78@gmail.com; 2 Department of Orthopaedic Surgery, CHA Bundang Medical Center, CHA University School of Medicine, 335 Pangyo-ro, Seongnam-si 13488, Gyeonggi-do, Republic of Korea; yks486ahj@naver.com (H.-J.A.); haeri1126@gmail.com (H.K.); insun10091@gmail.com (I.S.)

**Keywords:** osteoblast, osteoclast, supernatant, microfluidic, chip

## Abstract

We fabricated a microfluidic chip (osteoblast [OB]–osteoclast [OC] chip) that could regulate the mixture amounts of OB and OC supernatants to investigate the effect of different supernatant distributions on osteogenesis or osteoclastogenesis. Computer-aided design was used to produce an OB–OC chip from polydimethylsiloxane. A pressure controller was assembled and different blends of OB and OC supernatants were correctly determined. OB and OC supernatants were placed on the upper panels of the OB–OC chip after differentiation for an in vitro evaluation. We then tested the changes in osteogenesis using MC3T3-E1 cells in the middle chambers. We observed that a 75:25 distribution of OB and OC supernatants was the most potent in osteogenesis. We then primed the osteogenic differentiation of MC3T3-E1 cells using an OB–OC mixed supernatant or an OB supernatant alone (supernatant ratios of 75:25 or 100:0, respectively). These cells were placed on the calvarial defect sites of rats. Microcomputed tomography and histological analyses determined a significantly higher bone formation in the group exposed to the OB–OC supernatant at a ratio of 75:25. In this study, we demonstrate the applicability of an OB–OC chip to evaluate the effect of different supernatant distributions of OB and OC. We observed that the highest bone-forming potential was in MC3T3-E1 cells treated with conditioned media, specifically the OB–OC supernatant at a ratio of 75:25.

## 1. Introduction

Bone is highly dynamic tissue that is continuously remodeled to attain and maintain optimal bone integrity, mass, and strength. Bone homeostasis depends on the balance between the activity of osteoblasts (OBs) and osteoclasts (OCs). OBs are bone cells that primarily contribute to new bone formation. Their functions are essential to enhance the quality of bone [1,2,3]. OCs are the second key class of bone cells. They play a predominant role in the dissolution and absorption of old bone tissue, thereby contributing to essential bone maintenance, repair, and remodeling [4,5,6]. Resorption and formation are stable under physiological conditions, but bone architecture and its functions become abnormal when the balance is disturbed. An imbalance in this process can cause bone metabolism diseases such as osteoporosis or osteopetrosis [7]. Comprehending the mechanisms that regulate communication between OBs and OCs is critical when attempting to understand bone cell biology [8,9].

It is well known that osteoporosis and its associated fractures can be prevented by anti-resorption drugs [10]. Long-term treatment using these anti-resorptive drugs results in adverse events such as atypical fractures and osteonecrosis of the jaw because of the inhibition of bone remodeling from an excessive suppression of OCs [9]. These adverse events result in an increase in the resistance of patients with osteoporosis who are administered bisphosphonates in the long-term. There is, therefore, a requirement to develop new osteoporosis drugs [11]. Major advances in the understanding of the molecular mechanisms regulating OC functions have been achieved in the past 20 years, primarily from mouse and human genetic studies. These are the most basic but the most essential processes in bone remodeling and pathophysiology. Although it is widely known that the balance between OBs and OCs is important for the qualitative treatment of osteoporosis, there is minimal research on quantitative aspects such as the extent to which this balance contributes or the rate of contribution of OCs and OBs to bone formation.

We developed a 3D chip model to ascertain the most effective distribution of extracellular fluid through osteoblasts and osteoclasts to determine their quantitative contribution to bone formation. We attempted to confirm the results of bone formation by varying the ratio of extracellular fluid transported through osteoblasts and osteoclasts. We predicted that there would be an ideal ratio for osteoblast and osteoclast extracellular fluids that would result in the best bone formation. These results would be a measure of the contribution of osteoblasts and osteoclasts to bone formation.

The purposes of this study are to investigate the effects of different mixture proportions of OB and OC supernatants on bone formation and to determine the most effective ratio of OBs and OCs contributing to bone formation after ascertaining the most potent mixture ratio of OB and OC supernatants in bone formation.

## 2. Results

### 2.1. General Outline of OB-OC Chip Fabrication

**Figure 1:**
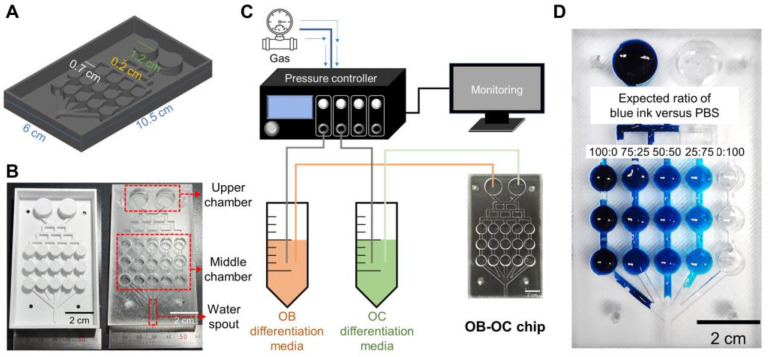
General outline of OB–OC chip fabrication. (**A**). A 3D model of the OB–OC chip. (**B**). OB–OC chip fabricated using polydimethylsiloxane. (**C**). Schematic diagram of the OB–OC chip and the assembly. (**D**). Concentration changes between blue ink and PBS according to the chamber location in the chip.

A 3D model of the OB–OC chip was designed with dimensions of 10.5 cm in length, 6 cm in width, and chamber diameters of 0.7 cm, 0.2 cm, and 1.2 cm (Figure 1A). The OB–OC chip was successfully fabricated using polydimethylsiloxane (PDMS). The chip featured designated upper chambers for seeding cells, middle chambers for media mixing, and waterspouts for fluid flow regulation (Figure 1B). The diagram in Figure 1 illustrates the assembly of the OB–OC chip, which had a pressure controller connected to differentiation media reservoirs for osteoblasts (OBs) and osteoclasts (OCs). This setup ensured precise control over the media flow and mixture ratios delivered to the chip (Figure 1C). Concentration changes in blue ink and phosphate-buffered saline (PBS) were observed according to the chamber location in the chip. This demonstrated the ability of the chip to create gradient mixtures of media. Expected ratios of 100:0, 75:25, 50:50, 25:75, and 0:100 were achieved, confirming the chip’s functionality in delivering precise mixtures of media to cultured cells (Figure 1D). These results validated the effectiveness of the OB–OC chip to generate specific media compositions for osteogenic studies.

### 2.2. Changes in Osteogenic Potential Using Different Percentages of OB–OC Supernatant Mixture

**Figure 2:**
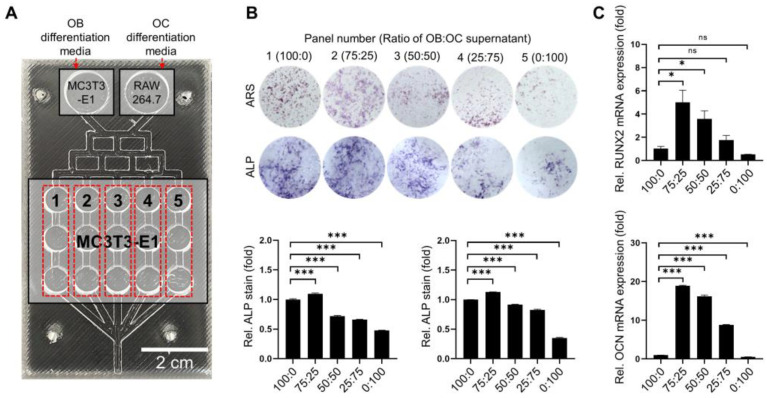
Effect of conditioned supernatant ratios on osteogenic differentiation. (**A**). Cell allocation of MC3T3-E1 and RAW 264.7 cells in the OB–OC chip. MC3T3-E1 cells were seeded in the middle chambers and exposed to five different OB–OC supernatant ratios (100:0, 75:25, 50:50, 25:75, and 0:100). Each middle chambers line is in triplication. (**B**). Representative images of alkaline phosphatase (ALP) and Alizarin Red S (ARS) staining, with quantification demonstrating the osteogenic differentiation of MC3T3-E1 cells under different OB–OC supernatant conditions. The quantification of the staining indicated that the 75:25 OB–OC supernatant ratio resulted in the highest osteogenic differentiation. Magnification: 40× (**C**). Quantitative reverse transcription polymerase chain reaction (qRT-PCR) results for the mRNA expression levels of osteogenic, Runt-related transcription factor 2 (RUNX2), and osteocalcin (OCN) markers in MC3T3-E1 cells treated using different OB–OC supernatant ratios. The 75:25 ratio significantly increased the expression of both RUNX2 and OCN compared with the other ratios, indicating enhanced osteogenic differentiation. A statistical significance is indicated as ns. *p* > 0.05, * *p* < 0.05, or *** *p* < 0.001.

MC3T3-E1 and RAW 264.7 cells were seeded on the upper left and right sides of the OB–OC chip, respectively. MC3T3-E1 cells were also seeded in the middle chambers. The middle chambers were supplemented with five different proportions of OB–OC supernatant (100:0, 75:25, 50:50, 25:75, and 0:100) (Figure 2A). The osteogenic differentiation potential of MC3T3-E1 cells in each conditioned medium was evaluated on day 7 using Alizarin Red S (ARS) and alkaline phosphatase (ALP) staining. The highest osteogenic differentiation was observed in cells treated with OB–OC media at a 75:25 ratio. Figure 2B,C illustrate the relative differentiation potential of each mixture. The quantitative reverse transcription polymerase chain reaction (qRT-PCR) results revealed that the expressions of osteogenic differentiation markers, Runt-related transcription factor 2 (RUNX2), and osteocalcin (OCN) were highest in cells treated with OB–OC media at a 75:25 ratio. These findings suggest that a certain amount of OC media stimulated osteogenic differentiation.

### 2.3. Changes in Osteoclastogenesis Using Different Percentages of OB–OC Supernatant Mixture

**Figure 3:**
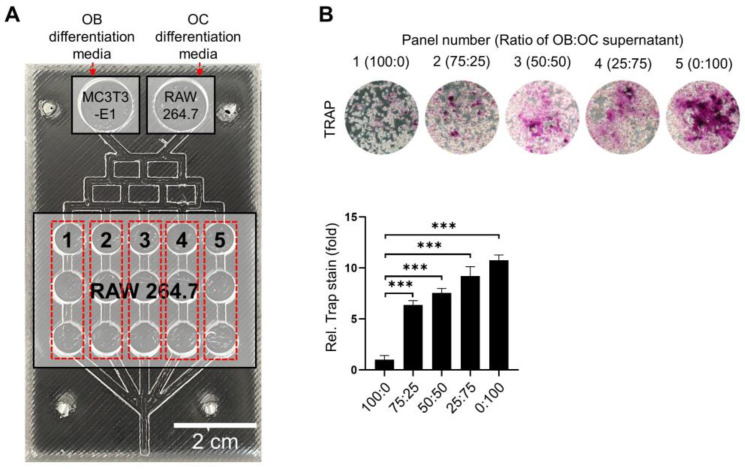
Effect of conditioned supernatant ratios on osteoclastic differentiation. (**A**). Cell allocation of MC3T3-E1 and RAW 264.7 cells in the OB–OC chip. RAW 264.7 cells were seeded in the middle chambers and exposed to five different OB–OC supernatant ratios (100:0, 75:25, 50:50, 25:75, and 0:100). Each middle chamber line is in triplication (**B**). Representative images of tartrate-resistant acid phosphatase (TRAP) staining with quantification demonstrating the osteoclastic differentiation of RAW 264.7 cells under different OB–OC supernatant conditions. The quantification of the staining indicated that the 0:100 OB–OC supernatant ratio resulted in the highest osteoclastic differentiation. A statistical significance is indicated as *** *p* < 0.001. Magnification: 40×.

MC3T3-E1 cells and RAW 264.7 cells were again seeded on the left and right sides of the upper chamber, respectively, but RAW 264.7 cells were placed in the middle chambers to evaluate the changes in OC differentiation under identical conditions. The RAW 264.7 cells were cultured for 5 days under different ratios of OB–OC supernatant (Figure 3A) and their OC activity was assessed using tartrate-resistant acid phosphatase (TRAP) staining. Unlike the effects observed in Figure 2, the OC differentiation gradually increased as the proportion of OB supernatant decreased from 100 to 0% (Figure 3B).

### 2.4. Changes in Osteogenic Differentiation of Related Genes in the 75:25 OB–OC Supernatant

**Figure 4:**
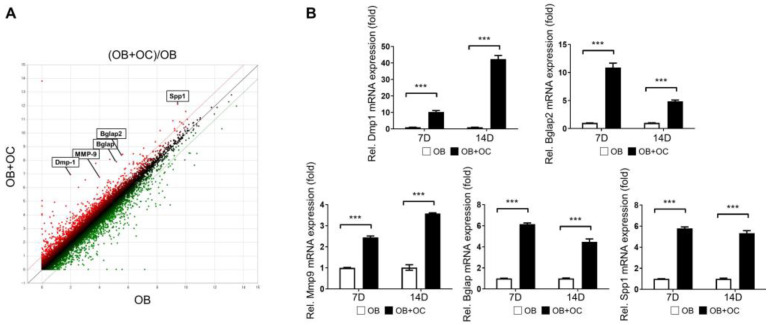
Changes in osteogenic-differentiation-related gene expression using an OB–OC supernatant ratio of 75:25. (**A**). Scatter plot of differentially expressed genes (DEGs) comparing gene expression levels between OB-only and OB+OC (75:25) conditions. Significantly Up and Down-regulated genes are filtered (|log 2 (Fold Change)| > 1, P adj < 0.05) and highlighted in red and green dots, respectively. The top five mRNA expressions related to osteogenesis (DMP1, MMP9, Bglap1, Bglap2, and SPP1) are highlighted. (**B**). Bar graphs depicting the changes in the mRNA expression levels of DMP1, MMP9, Bglap1, Bglap2, and SPP1 in MC3T3-E1 cells treated with OB only and the OB–OC supernatant at a 75:25 ratio over 7 and 14 days. A statistical significance is indicated as *** *p* < 0.001.

We conducted mRNA sequencing and performed a DEG analysis to investigate the biological functions influenced by the presence of OB+OC in osteogenesis. A scatter plot comparing OB with OB+OC revealed a notable presence of differentially expressed genes (DEGs), which were highlighted in red. Figure 4A demonstrates that the highlighted genes exhibited a fold-change of ≥2log2, presenting a statistical significance at *p* < 0.05. Specifically, the expressions of DMP1, MMP9, Bglap1, Bglap2, and SPP1 significantly changed (Figure 4A). The qRT-PCR results also validated the theory that the expression of the genes involved in osteogenic differentiation was significantly upregulated and promoted osteogenic differentiation (Figure 4B).

### 2.5. Higher Osteogenic Potential of the 75:25 OB-OC Supernatant Preconditioned with MC3T3-E1 Cells in a Critical Bone Defect Animal Model

**Figure 5:**
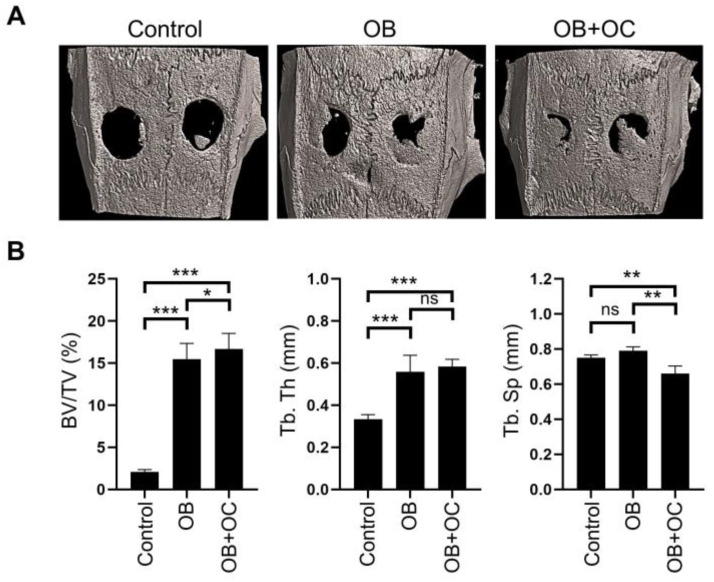
Microcomputed tomography analysis of calvarial defect healing. (**A**). Representative 3D microcomputed-tomography (micro-CT) images of calvarial defect sites in the control, OB, and OB+OC groups. The images illustrate the extent of bone regeneration in each group. (**B**). Bar graphs presenting the quantification of percent bone volume (BV/TV), trabecular thickness (Tb.Th), and trabecular separation (Tb.Sp) for each group. The OB+OC group demonstrated significantly higher BV/TV and Tb.Th compared with the control and OB groups, indicating enhanced bone formation. The OB+OC group also presented a reduced Tb.Sp, further evidencing improved bone regeneration. A statistical significance is indicated as ns. *p* > 0.05, * *p* < 0.05, ** *p* < 0.01, or *** *p* < 0.001.

We then assessed the osteogenesis-promoting effects of a 75:25 ratio of the OB–OC supernatant using a rat calvarial defect model. MC3T3-E1 cells were induced for osteogenesis in the developed OB–OC chip at a ratio of either 100:0 or 75:25 of the OB–OC supernatant for 7 days (Figure 1). Equal quantities of harvested cells (1 × 10^6^) were transplanted into rat calvaria. The representative 3D-reconstructed images of the calvaria are presented in Figure 5A. Quantitative analyses of microcomputed tomography (micro-CT) images were then conducted using the defect sites. Rats receiving the cells treated with the 75:25 OB–OC supernatant displayed a higher bone fraction volume (BV/TV) but lower trabecular separation (Tb.Sp) compared with rats receiving the cells treated with the 100:0 OB–OC supernatant (Figure 5B).

**Figure 6:**
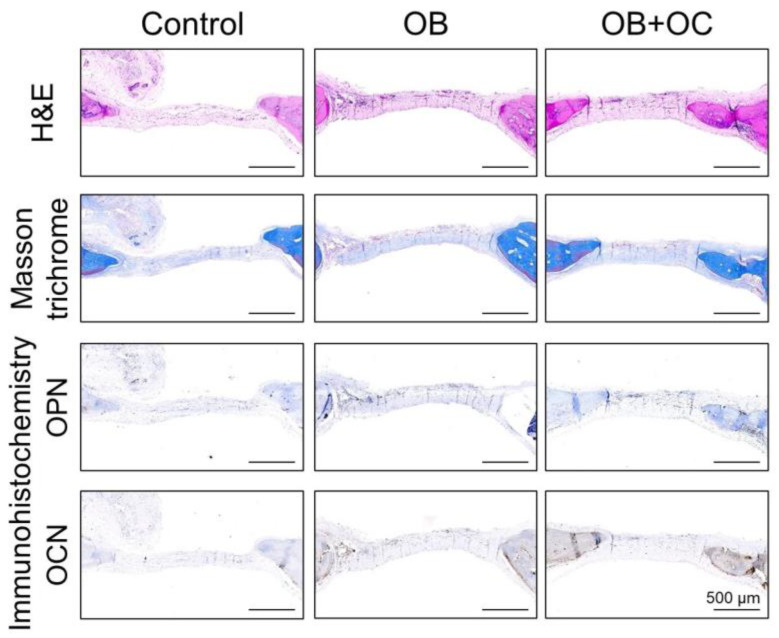
Histological analysis of bone regeneration. Representative histological images of calvarial defect sites from the control group, the OB (osteoblast) group, and the OB+OC (osteoblast+osteoclast) group. Hematoxylin and eosin staining presenting the overall tissue morphology and the extent of new bone formation. Masson’s trichrome staining illustrating collagen deposition and mature bone formation. Immunohistochemistry for osteopontin (OPN) and osteocalcin (OCN) demonstrating the expression levels of osteopontin and osteocalcin, which are the markers of bone formation and mineralization. Scale bar: 500 μm.

A precipitous cutoff was observed among the control-treated defects between the edge of the native parietal bone and adjacent fibrous tissue when using H&E and Masson’s trichrome staining. In contrast, the OB or OB+OC groups presented an increase in new-woven and lamellar bone formation. This was most prominent at the defect edge. The bone-forming effect was most noticeable in the high-dose OB+OC group, whereas the new bone formation was more subtle in the OB group (Figure 6). An immunohistochemical detection of the markers of bone healing was conducted. This included immunostaining for the osteogenic markers osteopontin (OPN) and osteocalcin (OCN). OPN and OCN immunohistochemistry decorated the OBs and osteocytes within the newly formed bone of the OB+OC group. Weak or an absence of staining for OPN and OCN was observed within the fibrous tissue associated with non-healing defects under control treatment conditions (Figure 6).

## 3. Discussion

The principal findings of this study were that the highest osteogenic differentiation potential was observed in cells treated with media conditioned with an OB–OC supernatant ratio of 75:25 and that a 3D-printed microfluidic chip could reproducibly distribute supernatant ratios of 100:0, 75:25, 50:50, 25:75, and 0:100 whilst effectively cultivating cells. We used an MC3T3-E1 cell culture to represent osteogenesis and an OB–OC supernatant distributed at ratios of 100:0, 75:25, 50:50, 25:75, and 0:100 on a 3D-printed microfluidic chip. The cells treated with conditioned media at an OB–OC supernatant ratio of 75:25 presented the most evident ARS and ALP staining and had the highest expressions of RUNX2 and OCN (known osteogenic differentiation markers) in the qRT-PCR results. It is well known that OCs play a predominant role in the dissolution and absorption of old bone tissue, thereby contributing to essential bone maintenance, repair, and remodeling [12]. However, there is insufficient knowledge of whether OCs can improve the bone-forming activity of OBs. Our study revealed that the supernatant from OCs contributed to the regulation of bone-forming activity to an extent.

There is increasing interest in the development of new in vitro models for bone-tissue engineering approaches. Microfluidic devices that incorporate a 3D architecture have been developed to study the vascularization, hematopoiesis, and cancer metastasis of bone [13,14,15,16,17]. We used 3D-printing technology to create an OB–OC chip for accurate and reproducible analyses where the media were evenly distributed during cell culture and differentiation. Our analyses confirmed that the supernatant at ratios of 100:0, 75:25, 50:50, 25:75, and 0:100 was reproducibly distributed and that the cells were well cultivated.

We created different media to ascertain the OB–OC supernatant conditions by manually mixing varying amounts of OB and OC supernatants one by one and not using the OB–OC chip. This was time-consuming and inaccurate in terms of the concentration control, although the results generally revealed similar trends. More accurate and faster results were obtained using the 3D-printed microfluidic chip.

The use of chip models is crucial when studying the relationship between bone formation and resorption for several reasons. Chip models can more accurately replicate an in vivo environment. This precision allows a more effective simulation of cell-to-cell interactions, mechanical stresses, and chemical signaling, which are essential when studying the dynamic relationship between bone formation and resorption. Bone formation and resorption are regulated by complex interactions between different cell types such as osteoblasts and osteoclasts. Chip models are ideal when studying how these cells interact in a 3D environment and how these interactions evolve over time. Chip models are valuable when modeling bone-related diseases such as osteoporosis and osteoarthritis and when testing new drug treatments. These models can provide results that are more relevant to human physiology compared with animal models. Chip models can integrate technologies for the real-time monitoring of bone formation and resorption processes. This enables researchers to observe and analyze cellular behaviors, signal transductions, and changes in the microenvironment as they happen. Chip models can be used to personalize medicine research. Researchers can study individual-specific bone regeneration and loss processes using a patient’s cells to analyze how specific treatments affect the patient. The use of chip models can reduce the need for animal testing, thus minimizing ethical concerns. This enhances the ethical standing of the research whilst providing results that have greater applicability to human biology.

Based on the results derived from the 3D-printed microfluidic chip model experiment, mRNA sequencing and DEG analyses were conducted using MC3T3-E1 cells cultured with an osteoblast-only supernatant and MC3T3-E1 cells cultured with an OB–OC supernatant at a ratio of 75:25. DMP1, MMP9, Bglap1, Bglap2, and SPP1—which are used as markers of osteogenic differentiation—were confirmed to be statistically significantly expressed in the OB–OC supernatant at a ratio of 75:25. MC3T3-E1 cells cultured with the osteoblast-only supernatant and MC3TC-E1 cells cultured with the OB–OC supernatant at a 75:25 ratio were transplanted onto rat calvaria in a rat calvarial defect model. Quantitative analyses of the micro-CT and histology results were conducted using several staining methods. Rats that received the cells treated with the 75:25 OB-OC supernatant presented a higher bone fraction volume (BT/TV) but lower trabecular separation (Tb.Sp) compared with the rats that received the cells treated with the OB-only supernatant. A significant and prominent bone-forming effect was demonstrated, even in the histology results, clearly revealing the role of OCs in osteogenic differentiation and affecting approximately 1/4 of the effect.

Xie et al. demonstrated that preosteoclasts, the immature progenitors of bone-degrading OCs, enhance angiogenesis and osteogenesis during bone remodeling through the release of platelet-derived growth factor B [18]. They investigated the role of OC-lineage cells in periosteal bone formation using mice lacking the gene encoding cytokine colony-stimulating factor 1, which is essential for the survival of cells in the monocyte/macrophage lineage. They observed that mice with an absence of cytokine colony-stimulating factor 1 had thin cortical bones and decreased bone vascularization. Liang et al. conducted a microarray analysis of small extracellular vesicles and microRNAs secreted from OCs at different stages. They identified four miRNAs that were highly expressed in mature OC-derived serum extracellular vesicles [19]. One of these miRNAs, miR-324, significantly induced the OB differentiation and mineralization of primary mesenchymal stem cells in vitro by targeting ARHGAP-1, a negative regulator of OB differentiation. In our study, we confirmed that the supernatant from OCs contributed to osteogenesis and that a ratio of 75:25 (OB:OC) presented superior results for bone-forming markers as well as for actual bone forming than cells only differentiated using commercial media.

There were a few limitations to this study. First, we did not perform an experiment regarding the mechanism of action; for example, which substance in the OC supernatant reinforced the osteogenic differentiation of MC3T3-E1 cells. Second, we only investigated the indirect effect of the cells on osteogenesis or osteoclastogenesis. Third, we could only use five different ratios of mixture in the OB–OC chip. Developing an advanced microfluidic chip is necessary to evaluate the effect of OBs and OCs with a direct attachment to a fine mixture control.

In conclusion, our study demonstrated the efficiency of a novel OB–OC culture chip for the different distribution of supernatants. The primary result was that the highest osteogenic differentiation potential was observed in cells treated with a conditioned supernatant at an OB:OC ratio of 75:25.

## 4. Materials and Methods

### 4.1. OB–OC Chip Fabrication and Accessory Assembly

A mold to mix the OB and OC supernatants was designed and fabricated using a 3D printer. The exact dimensions of the entire chip and its holes and channels are specified in Figure 1A. The chip consisted of two types of chamber (upper and middle) and waterspouts. The upper and middle chambers had 2 and 15 holes, respectively, for cell allocation. The OB–OC chip was fabricated using polydimethylsiloxane. A photograph of an optical chip fabricated using the 3D-printed mold is presented in Figure 1B. A computer-connected Elveflow OB1 MK3 (Elveflow, Paris, France) pressure controller was assembled to regulate the pressure in each channel and to ensure a continuous supernatant flow. Microfluidic chip flow conditions are shown in Table 1.The drained supernatant was discarded into a bucket connected to the waterspouts. The experimental conditions used in this study are schematically shown in Figure 1C, which also demonstrates how the solution flowed out of each hole and blended as it proceeded down the channel. Blending was initially evaluated using blue ink and phosphate-buffered saline (PBS) (Figure 1D).

### 4.2. Cell Allocation

We used MC3T3-E1 cells and RAW 264.7 cells for the osteogenesis and osteoclastogenesis analyses, respectively. We placed the MC3T3-E1 cells and RAW 264.7 cells in the right and left sides of the upper chambers after inducing the OB and OC differentiation. We then located the MC3T3-E1 cells or RAW 264.7 cells in the middle chambers for the evaluation of osteogenesis changes (Figure 1) or osteoclastogenesis changes (Figure 2). Details of the culture and differentiation protocol are described below.

### 4.3. In Vitro Cell Culture and Differentiation

MC3T3-E1 cells were cultured in an α-minimum essential medium supplemented with 10% (*v*/*v*) fetal bovine serum (FBS), 0.22% sodium bicarbonate, 100 U/mL penicillin, and 100 μg/mL streptomycin at 37 °C in a humidified atmosphere containing 95% air and 5% CO_2_. Osteogenesis differentiation was initiated when a cell confluence was established with 50 μg/mL ascorbic acid and 10 mM β-glycerophosphate in a complete cell culture medium for the indicated periods. The medium was continuously propelled using a pressure controller (Figure 1C). After 7 days of osteogenic differentiation, the cells were fixed in 4% paraformaldehyde for 10 min. Alkaline phosphatase (ALP) staining was performed using an NBT/BCIP staining kit, following the manufacturer’s instructions. ALP activity was determined at 405 nm using a reaction buffer and p-nitrophenyl phosphate as a substrate. This was calculated after normalization to the total protein content. The protein content was measured using the bicinchoninic acid (BCA) method with a Pierce protein assay kit (Thermo Fisher Scientific, Waltham, MA, USA). Mineralized nodule formation was determined using ARS staining. Briefly, the cells were fixed in 95% ethanol for 20 min at room temperature, washed with distilled water, and finally stained with 0.1% ARS (pH = 4.2; Sigma-Aldrich, St. Louis, MO, USA) for 30 min after osteogenic incubation for the indicated days. The stain was dissolved in cetylpyridinium chloride (Sigma-Aldrich, USA) and the absorbance at 570 nm was measured to quantitatively assess the degree of mineralization.

RAW264.7 cells were acquired from the American Type Culture Collection (ATCC, Manassas, VA, USA). The culture condition of the cells was 10% (*v*/*v*) FBS, 0.22% sodium bicarbonate, 100 U/mL penicillin, and 100 μg/mL streptomycin added into Dulbecco’s modified eagle medium (DMEM). Macrophage colony-stimulating factor (50 ng/mL) and the receptor activator of the nuclear factor κ-B ligand (50 ng/mL) were used to continuously treat the RAW 264.7 cells for 5 days. A pressure controller was used to ascertain osteoclastogenesis. The cell supernatant was then discarded. The cells were fixed for 5 min and then stained using TRAP.

### 4.4. RNA Isolation and qRT-PCR Analyses

Total RNA was isolated from cultured cells or frozen tumor tissues using a TRIzol^®^ reagent (Invitrogen; Thermo Fisher Scientific, USA) or an RNeasy mini kit (Qiagen GmbH, Hilden, Germany), respectively, according to the manufacturers’ protocols. The total RNA (1 µg) was reverse transcribed into cDNA using an AMPIGENE^®^ cDNA synthesis kit (Enzo Biochem, Farmingdale, NY, USA) to quantify the mRNA expression. The reaction mixtures were sequentially incubated at 42 °C for 30 min and at 85 °C for 10 min. The qPCR for the mRNA determination was conducted using AMPIGENE^®^ qPCR Green Mix (Enzo Biochem, USA) and an iCycler real-time PCR detection system (Bio-Rad Laboratories, Hercules, CA, USA), according to the manufacturers’ protocols. The qPCR reaction conditions were denaturation at 95 °C for 2 min, followed by 40 cycles at 95 °C for 5 s, 60 °C for 30 s, and 72 °C for 30 s. β-Actin was used as the endogenous control for the mRNA determination. The sequences of the specific primers for the qRT-PCR analyses of mRNAs are listed in Table 2.

### 4.5. mRNA Sequencing

We conducted the library preparation and sequencing of the total RNA of the MC3T3-E1 cells to validate the alterations in the mRNA expression of the MC3T3-E1 cells throughout the osteogenesis process using different ratios of the OB–OC supernatant. These cells were differentiated with either a 100% OB supernatant or a 75:25 ratio of the OB–OC supernatant. We used a QuantSeq 3′ mRNA-Seq Library Prep Kit (Lexogen, Inc., Vienna, Austria), following the manufacturer’s protocol. Initially, 500 ng of total RNA per sample was processed. An oligo-dT primer incorporating an Illumina-compatible sequence at its 5’ end was employed for RNA hybridization, followed by reverse transcription. Subsequently, RNA template degradation and a second-strand synthesis were initiated using a random primer with an Illumina-compatible linker sequence. The resulting double-stranded libraries were purified using magnetic beads to eliminate the residual reaction components. Library amplification was conducted to incorporate the complete adapter sequences necessary for the cluster generation. The final libraries underwent purification to remove the PCR components. High-throughput sequencing was performed as single-end 75 sequencing using a NextSeq 500 platform (Illumina, Inc., San Diego, CA, USA). QuantSeq 3′ mRNA-Seq reads were aligned using Bowtie2. Bowtie2 indices were generated from either the genome assembly sequence or representative transcript sequence to align with the genome and transcriptome. The resulting alignment files were used for the transcript assembly, abundance estimation, and identification of differentially expressed genes. A DEG analysis was conducted based on the unique and multiple alignment counts. We used Bedtools for coverage. Read count (RC) data were processed via quantile normalization using EdgeR within R (R Development Core Team, 2016) with Bioconductor. Data mining and graphical visualizations were executed using ExDEGA graphicplus (v 2.0) (Ebiogen Inc., Seoul, Republic of Korea).

### 4.6. Rat Calvarial Defect Model

MC3T3-E1 cells were induced under different conditions for 7 days and then divided into three groups. These were the control group, comprising only PBS; the OB group, comprising MC3T3-E1 cells treated with OB differentiation media and 100% OB supernatant; and the OB+OC group, comprising MC3T3-E1 cells incubated with OB differentiation media and 75:25 OB-OC media. We used Sprague Dawley rats, which were handled in compliance with the guidelines for the care and use of animals at our institution. The rats were maintained under specific pathogen-free conditions at 23 ± 1 °C with 50 ± 10% humidity and a 12/12 h light/dark cycle. Food and water were provided ad libitum. The animal procedures were approved by the Institutional Animal Care and Use Committee (IACUC) of the CHA Laboratory Animal Research Center (IACUC230118). The methods used followed the approved guidelines and all efforts were taken to minimize the number of animals used and their suffering. Rats aged 8 weeks were randomly assigned to three groups of five each. A circular and full-thickness bone defect with a diameter of 4 mm was produced on both parietal bones using a burr. PBS or cells were adsorbed onto an absorbable collagen sponge (Advanced BioMatrix, San Diego, CA, USA), which was fabricated with a diameter of 4 mm. The chemically adsorbed collagen sponges were placed in the calvaria region of the rats and the defects were covered with the pericranium. The rats were sacrificed eight weeks after the operation. All the calvaria were dissected and fixed in 10% buffered paraformaldehyde.

### 4.7. Micro-CT Analyses

The specimens were scanned using a micro-CT imaging system (Quantum GX; PerkinElmer, Hopkinton, MA, USA) at an isotropic resolution of 40 μm. The images of each sample were quantitatively measured to ascertain the amount of newly formed hard tissue in the pulp space using 3D Slicer 4.11.0 software (https://www.slicer.org accessed on 30 January 2022). Bone regeneration in the rat calvarial defect areas was evaluated and visualized using a high-resolution Skyscan 1173 Micro-CT system (Bruker, Aartselaar, Belgium). Images were acquired at an effective pixel size of 6.04 μm, a voltage of 130 kV, a current of 60 μA, and an exposure time of 500 ms. Image reconstruction software (NRecon v1.17.7; Bruker) was used to reconstruct serial cross-sectional images with identical thresholds for all samples (0–6000 in Hounsfield units). The BV/TV, Tb.Th, and Tb.Sp rates were examined using CTAn software v.1.20.8 (Bruker Micro-CT, Billerica, MA, USA). The 3D rat calvarial micro-CT images were obtained using CTvox software v.3.3.1 (Bruker Micro-CT, USA).

### 4.8. Histological Analyses

The rat calvaria were decalcified using 10% ethylenediaminetetraacetic acid (EDTA; pH = 7.4) for 1 month. The EDTA solution was replaced every 2 days. The decalcified samples were washed, dehydrated, and embedded in paraffin. The paraffin-embedded sections were prepared, dewaxed, and rehydrated. Sections of 5 μm were cut and stained using standard hematoxylin and eosin (H&E) and Masson’s Trichrome staining.

Additional sections were incubated with the following primary antibodies to ascertain the immunohistochemistry: anti-OPN (sc-21742; 1/100; Santa Cruz Biotechnology, Dallas, TX, USA), anti-OCN (sc-390877; 1/100; Santa Cruz Biotechnology), and anti-MMP-9 (sc-7269; 1/100; Santa Cruz Biotechnology). The following secondary antibodies were used: biotinylated anti-rabbit or anti-goat IgG secondary antibody (1/200; Dako, Groschup, Denmark). The paraffin slices were deparaffinized, dehydrated, rinsed, and incubated with 3% H_2_O_2_ for 20 min using the ABC method (Vector Laboratories, Inc., Newark, CA, USA). Trypsin-induced epitope retrieval was performed for 20 min at room temperature using a Digest-All 2 system (Cat 00-3008; Invitrogen, Waltham, MA, USA). All sections were then blocked with 0.1% bovine serum albumin in PBS for 1 h. Primary antibodies were added to each section at their respective dilutions and incubated at 37 °C for 1 h or overnight at 4 °C. Positive immunoreactivity was detected following the ABC complex (PK-6100, Vectastain Elite ABC Kit; Vector Laboratories, Inc.) Incubation and development were ascertained using AEC chromagen (K346911-2; Dako). Mayer’s hematoxylin (1/5; Abcam, Cambridge, UK) was used as a nuclear counterstain. The slides were mounted using aqueous media (VectaMount AQ, Vector Laboratories).

The staining intensity of the immunohistochemically stained cross-section images was evaluated using Image J software (v. 1.42l) and light microscopy. Analyses were conducted using images obtained from 6 to 12 adjacent high-power fields to encompass the entire defect site of each animal on a representative mid-defect section.

### 4.9. Statistical Analyses

All data were expressed as the mean ± standard error of three independent experiments. All statistical analyses were conducted using GraphPad Prism software, version 8 (GraphPad Software, San Diego, CA, USA). The comparison of the two groups was conducted using Student’s *t*-test. A one-way ANOVA with Tukey’s post hoc test was used for the comparison of more than two groups. A *p*-value < 0.05 was considered to be a statistically significant difference.

## Figures and Tables

**Table 1:** Microfluidic chip flow conditions.

MFS Flow Sensors	MFS2
Media calibration	Water/aqueous solutions
Flow-rate range	0 to ±7 μL/min
Accuracy Measured value (m.v.) applies to negative values (bi-directional)	20 nL/min between 0 and 0.42 μL/min
	5% m.v. between 0.42 and 7 μL/min
Repeatability Measured value (m.v.) applies to negative values (bi-directional)	3.5 nL/min between 0 and 0.7 μL/min
	0.5% m.v. between 0.7 and 7 μL/min
Pressure drop at full-scale flow rate, 23 °C	3 mbar
Total internal volume	1.5 μL
Sensor inner diameter	150 μm
Tubing inner length	29 mm
Operating pressure	200 bar
Burst pressure	400 bar
Microfluidic fitting type	UNF 1/4–28
Wetted material	PEEK
Internal sensor capillary material	Quartz

**Table 2:** List of primers.

Gene	Sequence (5′-3′)		Tm
Dmp1	Forward	AAAGACCACGACAGTGAGGAT	60.8
	Reverse	CATCATCGAACTCAGAACCGTC	60.7
Bglap2	Forward	CTGACCTCACAGATCCCAAGC	62.1
	Reverse	TGGTCTGATAGCTCGTCACAAG	61.5
Mmp9	Forward	GCAGAGGCATACTTGTACCG	60.2
	Reverse	TGATGTTATGATGGTCCCACTTG	60.0
Bglap	Forward	AAGCAGGAGGGCAATAAGGT	60.1
	Reverse	TTTGTAGGCGGTCTTCAAGC	60.4
SPP1	Forward	ATCTCACCATTCGGATGAGTCT	60.4
	Reverse	TGTAGGGACGATTGGAGTGAAA	60.8
RUNX2	Forward	CCAACCGAGTCATTTAAGGCT	57.3
	Reverse	GCTCACGTCGCTCATCTTG	58.1
OCN	Forward	CTGACCTCACAGATCCCAAGC	59.4
	Reverse	TGGTCTGATAGCTCGTCACAAG	58.9

## Data Availability

The original contributions presented in the study are included in the article, further inquiries can be directed to the corresponding author.
